# Black Cumin Seed (*Nigella sativa*) Confers Anti‐Adipogenic Effects in 3T3‐L1 Cellular Model and Lipid‐Lowering Properties in Human Subjects

**DOI:** 10.1002/fsn3.70888

**Published:** 2025-09-01

**Authors:** Shamima Ahmed, Mohammad Shaokat Ali, Yuki Nishigaki, Ranita Das, Sumsuddin Ahmed Shiblu, Sharmin Akter, Isao Matsui‐Yuasa, Akiko Kojima‐Yuasa

**Affiliations:** ^1^ Department of Nutrition, Graduate School of Human Life and Ecology Osaka Metropolitan University Osaka Japan; ^2^ Faculty of Food Science and Technology Chattogram Veterinary and Animal Sciences University Chattogram Bangladesh; ^3^ Chittagong Medical College Hospital Chattogram Bangladesh; ^4^ Karnafully Diabetic Center, Osman Mansion Chattogram Bangladesh

**Keywords:** adipogenesis, black cumin seed, lipid‐lowering properties, obesity

## Abstract

*Nigella sativa* (black cumin seed) has traditionally been valued for its medicinal properties. This study explored its potential in addressing obesity‐related conditions by assessing its anti‐adipogenic and lipid‐lowering effects. Black cumin seed extract showed high phenolic (35.48 mg GAE/g DW) and flavonoid (39.51 mg QE/g DW) contents with excellent standard curve linearity (*R* ^2^ > 0.99). FTIR confirmed thymoquinone‐related functional groups, and GC–MS revealed 23 fatty acids, predominantly methyl eicosatrienoate (69.29%), methyl 11,14,17‐eicosatrienoate (25.2%), and methyl linoleate (4.05%). These results indicate a rich phytochemical and fatty acid profile. In vitro, 3T3‐L1 preadipocytes were treated with a methanolic black cumin seed extract (BSE). Oil red O staining revealed a significant reduction in lipid accumulation, while cell viability assays confirmed no cytotoxicity. Gene expression analysis demonstrated a marked downregulation of key adipogenic transcription factors, including C/EBPα, C/EBPβ, and PPARγ, following BSE treatment. A randomized controlled trial (RCT) further evaluated its effects in humans. Participants in the test group consumed 5 g of black cumin seed powder daily for 8 weeks, while the control group received no supplementation. Appetite levels were monitored using the Council on Nutrition Appetite Questionnaire (CNAQ), with reliability ensured through Cronbach's alpha validation. Serum lipid profiles, including triglycerides (TG), low‐density lipoprotein cholesterol (LDL‐C), high‐density lipoprotein cholesterol (HDL‐C), and total cholesterol (TC), were assessed pre‐ and post‐intervention. Results indicated that the black cumin seed group exhibited statistically significant reductions in TG, LDL‐C, and TC levels, alongside an increase in HDL‐C, while the control group showed no notable reductions. Our findings suggest that black cumin seed may offer potential anti‐adipogenic and lipid‐lowering benefits, contributing to obesity management.

## Graphical abstract

Black cumin seed extract (BSE) reduced lipid accumulation of 3T3‐L1 preadipocytes without cytotoxicity. BSE downregulated the gene expression of key adipogenic transcription factors, including C/EBPα, C/EBPβ, and PPARγ. Black cumin seed exhibited significant reductions in serum TC, TG, and LDL‐C levels in humans.

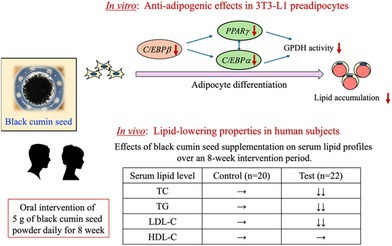

## 1 Introduction

Obesity is a global health concern associated with metabolic disorders such as insulin resistance, dyslipidemia, and cardiovascular diseases (Ginsberg 2000; Powell‐Wiley et al. 2021). The process of adipogenesis, which involves the differentiation of preadipocytes into mature adipocytes, plays a crucial role in the development of obesity and related complications. Several transcription factors, including peroxisome proliferator‐activated receptor gamma (PPARγ) and CCAAT/enhancer‐binding proteins (C/EBPs), regulate adipogenesis and lipid accumulation (Rosen et al. 2002).

*Nigella sativa* , or black cumin, is a flowering plant from the *Ranunculaceae* family long used in traditional medicine across South Asia, North Africa, and the Mediterranean. Its therapeutic properties are recognized in ancient systems like Unani, Ayurveda, and Tibb (Mohebbati and Abbasnezhad 2020). Black cumin seed and its oil are rich in bioactive compounds such as alkaloids, flavonoids, and essential oils, contributing to their broad pharmacological potential (Ahmad et al. 2021). Their diverse physicochemical properties make them valuable in both food and medicine. Cell culture and animal studies have demonstrated the therapeutic effects of black cumin and its active constituent, thymoquinone (TQ), including antimicrobial, anti‐inflammatory, antioxidant, antidiabetic, antihypertensive, antitumor, immunomodulatory, and anti‐obesity activities (Ahmad et al. 2021; Bashir et al. 2023; Bhavikatti et al. 2024). Anticancer effects of black cumin or TQ have been reported in various cancer cell lines and animal models (Homayoonfal et al. 2022; Zheng et al. 2016). TQ also inhibits adipogenesis through pathways such as AMPK and MAPKs (Ahmed et al. 2025; Ramineedu et al. 2024). However, the molecular mechanisms behind its anti‐adipogenic action remain incompletely understood.

Black cumin seed has been investigated for its effects on serum cholesterol levels in humans. Studies have shown that interventions using the seed powder or oil can significantly improve lipid profiles. It helps lower total cholesterol, LDL (low‐density lipoprotein) cholesterol, and triglycerides, while simultaneously increasing HDL (high‐density lipoprotein) cholesterol levels (Uma Maheswari et al. 2022). Clinical trials have demonstrated that regular consumption of black cumin seed at doses of 500 mg to 2 g per day over a few weeks results in favorable cholesterol modulation. These effects are particularly beneficial for individuals with hypercholesterolemia or other cardiovascular risk factors (Hosseinzadeh et al. 2017; Sahebkar et al. 2016).

In this study, we investigated the anti‐adipogenic potential of black cumin seed extract (BSE) in the 3T3‐L1 cellular model by assessing cell viability using the neutral red assay, lipid accumulation through oil red O staining, and adipogenic gene expression via real‐time PCR. Additionally, we conducted a randomized controlled human trial to evaluate the lipid‐lowering properties of black cumin seed powder by measuring serum cholesterol levels. To assess the potential impact on appetite, we utilized the Council on Nutrition Appetite Questionnaire (CNAQ), while the reliability of the assessment was verified using Cronbach's alpha coefficient (Hanisah et al. 2012; Wilson et al. 2005).

Our findings offer important insights into the potential use of black cumin seed as a natural intervention for obesity. By combining in vitro and clinical evidence, this study demonstrates the ability of black cumin seed to regulate lipid metabolism and inhibit adipogenesis, all while not negatively affecting participant's appetite. This research adds to the expanding body of research on functional foods for improving metabolic health.

## 2 Materials and Methods

### 2.1 Collection of Black Seeds

Black cumin seeds ( *Nigella sativa* , Indian Kalonji variety) were purchased from Amazon Japan, supplied by Kobe RT Spices (Japan), with species identity specified by the supplier. The seeds were thoroughly washed with ample water to remove any adhering impurities and then dried (40°C for 24 h) using a cabinet dryer. Subsequently, the seeds were ground into powder using a grinder and stored in sealed containers for future research.

### 2.2 Materials of Cell Culture

Dulbecco's Modified Eagle Medium (DMEM) from Shimadzu Diagnostics Corporation, fetal bovine serum (FBS) from Sigma‐Aldrich Japan LLC, penicillin potassium and streptomycin sulfate from Meiji Seika Pharma Co. Ltd., and a few other products from FUJIFILM Wako Pure Chemical Co. Ltd., such as dimethyl sulfoxide (DMSO), Neutral Red, Oil Red O, 2‐Propanol, and 2‐Mercaptoethanol, as well as dihydroxyacetone phosphate dilithium salt from Sigma‐Aldrich Japan LLC.

### 2.3 Extraction of Black Cumin Seed

Black cumin seed was meticulously cleaned and dried at 50°C overnight using a hot air oven (Yamato Drying Sterilizer, SH‐41, Yamato Scientific Co. Ltd., Japan) before being finely milled using a grinder (ZOJIRUSHI BM‐KA04‐GS, China). The resulting powder was stored in an airtight, light‐protected container until needed. Extraction was carried out using the methanolic extraction method with a rotary evaporator (EYELA N‐1000, Tokyo Rikakikai Co. Ltd., Japan).

### 2.4 Phytochemicals in Seed Extract

Total phenolic content (TPC) was determined using the Folin–Ciocalteu method (Singleton and Rossi 1965), where 1 mL of extract or gallic acid standard (2–32 μg/mL) was mixed with diluted Folin–Ciocalteu reagent and sodium carbonate, incubated for 60 min, and absorbance measured at 765 nm using a UV–visible spectrophotometer (UVD‐3000, Labomed, USA). TPC was expressed as mg gallic acid equivalent per gram dry weight (mg GAE/g DW). Total flavonoid content (TFC) was measured by the aluminum chloride colorimetric assay (Chang et al. 2002), where 1 mL of extract or quercetin standard (6–96 μg/mL) was reacted with AlCl_3_, potassium acetate, and water, incubated for 30 min, and absorbance measured at 420 nm. TFC was expressed as mg quercetin equivalent per gram dry weight (mg QE/g DW). All measurements were performed in triplicate, and values were reported as mean ± standard deviation.

### 2.5 FTIR Spectroscopic Analysis of Seed Extract

Fourier Transform Infrared (FTIR) spectroscopy was performed to identify the functional groups present in the methanolic extract of black cumin seed using an FTIR spectrometer (Perkin Elmer Spectrum II, USA). A small amount of the dried extract was mixed with spectroscopic‐grade potassium bromide (KBr) and compressed into a transparent pellet under hydraulic pressure. The spectra were recorded in the range of 4000–400 cm^−1^ with a resolution of 4 cm^−1^ and 32 scans per sample.

### 2.6 Fatty Acid Profile by GC–MS

Fatty acid profiling of black cumin seed was performed using GC–MS (QP‐2020, Shimadzu, Japan). Seeds were cleaned, dried, ground, and lipids were extracted using Soxhlet extraction with n‐hexane. The oil was concentrated via rotary evaporation and stored at 4°C. For FAME preparation, 100 mg of oil was mixed with 2 mL of 0.5 M methanolic KOH and heated at 60°C for 30 min, followed by the addition of 2 mL of n‐hexane. The resulting organic layer was filtered (0.22 μm) before GC–MS analysis. FAMEs were analyzed using an Agilent 7890A GC coupled with a 5975C MS detector and an HP‐5MS capillary column (30 m × 0.25 mm × 0.25 μm). Injection was at 250°C (1 μL, split 10:1), with helium as the carrier gas at 1.0 mL/min. The oven was programmed from 60°C to 280°C with specified ramping. Mass spectra were recorded in EI mode (70 eV, m/z 40–550). Fatty acids were identified using FAME standards and the NIST library, quantified by area normalization, and expressed in ppm and percentage of total fatty acids. All analyses were conducted in triplicate, and results are presented as mean ± SD.

### 2.7 Cell Culture

The 3T3‐L1 preadipocytes (JCRB9014) were sourced from the Japanese Cancer Research Resources Bank. These cells were initially cultured in DMEM with 10% FBS. Upon reaching confluence, adipocyte differentiation was induced using a mixture of 0.25 μM dexamethasone, 0.5 mM 3‐isobutyl‐1‐methylxanthine, and 0.2 μM insulin (DMI) in DMEM with 10% FBS. The cells were subsequently cultured for 2 days in DMEM containing 10% FBS and 0.2 μM insulin, followed by an additional 4 days in DMEM with 10% FBS. A methanolic extract of BSE was dissolved in DMSO, with the final concentration of DMSO in the medium being kept below 0.5%. Control cultures comprising cells, media, and DMSO were prepared for each experiment.

### 2.8 Cell Viability

The neutral red assay, as described by Riddell et al. (1986), was employed to assess cell viability. After culturing, cells were incubated at 37°C for 2 h, followed by the addition of neutral red reagent to a final concentration of 50 μg/mL. Post‐incubation, the cells were cleansed with 2 mL of a 1% formaldehyde and 1% CaCl_2_ solution, along with 1 mL of a 1% CH_3_COOH and 50% ethanol solution, and allowed to sit at room temperature for 30 min. The absorbance of the extract was then measured at 540 nm using a JASCO V‐730 BIO Spectrophotometer (JASCO Corporation, Japan). The following formula was used to measure cell viability:

$$\begin{array}{c} \text{Viability}\;\left(\%\right)=\frac{A540-\text{treated cells}}{A540\;\text{of appropriate control}}\\ \times 100\;\text{after correction for background absorbance} \end{array}$$

### 2.9 Oil Red O Stain

During the differentiation process into mature adipocytes, oil red O staining was used to evaluate lipid accumulation (RamírezZacarías et al. 1992). A 2 mL wash with Ca^++^ and Mg^++^ free‐phosphate buffer saline (PBS (−)) was carried out following the aspiration of the media from 3T3‐L1 adipocytes. After 60% ethanol was used for fixation, 1 mL of oil red O staining solution was added, and the mixture was allowed to stand for 30 min. One milliliter of 2‐propanol was used for extraction following two cleanings with one milliliter of ultrapure water and a 50% ethanol wash. With a spectrophotometer (JASCO V‐730 BIO Spectrophotometer, JASCO Corporation), the absorbance of the extract was measured at 520 nm in wavelength.

### 2.10 Glycerol‐3‐Phosphate Dehydrogenase (GPDH) Activity

Adipocytes 3T3‐L1 were obtained 8 days following the initiation of differentiation. The cells underwent two 1 mL PBS (−) washes. Harvesting involved using a cell scraper and 350 μL of triethanolamine/EDTA buffer, followed by cell disruption using a sonicator (Bioruptor UCD‐250, COSMO BIO Co. LTD, Japan). After centrifugation (13,000 × *g*, 5 min, 4°C), the resulting supernatant underwent an enzyme test. GPDH activity was assessed using the Wise and Green method (Wise and Green 1979). Enzyme activity was calculated by monitoring the change in NADH over 3 min, using the extinction coefficient of nicotinamide adenine dinucleotide, which is 6.22 mM^−1^ cm^−1^. The enzyme activity was expressed as a percentage relative to a control set at 100%.

### 2.11 Quantitative Reverse Transcription‐Polymerase Chain Reaction (qRT‐PCR)

RNA was isolated from 3T3‐L1 adipocytes using the High Pure RNA Isolation Kit (Roche, Germany). The integrity and concentration of the extracted RNA were assessed with the 2100 Bioanalyzer (Agilent Technology Inc.). Complementary DNA (cDNA) synthesis was performed using the PrimeScript RT Reagent Kit (TaKaRa Bio Inc., Japan). Subsequently, real‐time PCR was conducted on a StepOnePlus PCR System (Thermo Fisher Scientific Inc.) employing TB Green Premix Ex Taq II (TaKaRa Bio Inc.) in accordance with the manufacturer's protocols. Primer sequences are provided in Table 1. Messenger RNA (mRNA) expression levels were normalized to β‐actin, and relative quantification was carried out using the delta–delta CT method with StepOne software version 2.2.2 (Thermo Fisher Scientific Inc.).

**TABLE 1:** The sequences of primes used in real‐time PCR.

Name	Forward	Reverse
*C/EBPα*	5′‐TTGAAGCACAATCGATCCATCC‐3′	5′‐GCACACTGCCATTGCACAAG‐3′
*C/EBPβ*	5′‐ACGGGACTGACGCAACACA‐3′	5′‐TGCTCGAAACGGAAAAGGTTC‐3′
*PPARγ*	5′‐GGAGCCTAAGTTTGAGTTTGCTGTG‐3′	5′‐TGCAGCAGGTTGTCTTGGATG‐3′

### 2.12 Design of Research and Phenomena of Participants

**FIGURE 1:**
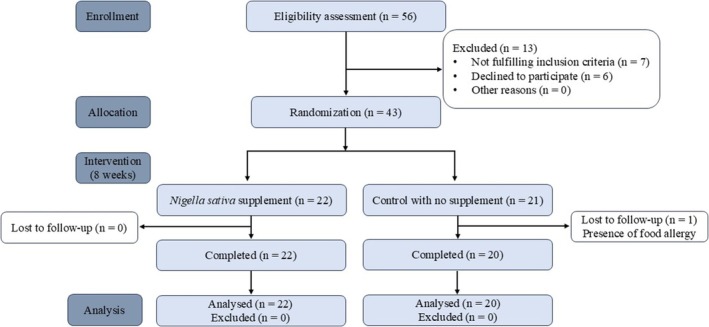
CONSORT 2010 flow diagram for clinical trial on black cumin seed powder intervention. The diagram illustrates the flow of participants through the randomized clinical trial evaluating the effects of black cumin seed powder intervention. Participants were screened for eligibility, and those meeting the inclusion criteria were randomly assigned into two groups: The test group (receiving black cumin seed powder) and the control group (not receiving black cumin seed powder). The flowchart details the number of participants allocated to each group, those who completed the intervention, and any losses to follow‐up or exclusions. The final analysis includes participants who completed the study per protocol.

This investigation was conducted using a randomized controlled clinical trial approach. Our study adhered to the guidelines outlined in the CONSORT 2010 Statement for reporting randomized controlled trials. The study was conducted at the Karnafully Diabetic Centre in collaboration with Chittagong Medical College Hospital. Figure 1 outlines the human clinical trial protocol. The sample size for the clinical trial was estimated using G*Power software (version 3.1.9.7; Heinrich Heine University, Düsseldorf, Germany), following the method described by Kang (2021). An a priori power analysis was conducted based on a two‐tailed independent samples *t*‐test to compare the mean change in total cholesterol levels between the intervention group (black cumin seed powder) and the control group. Participants were selected from patients with borderline and high cholesterol levels who were not undergoing medication. Eligible participants included those who were willing and satisfied to join the study and did not have specific medical conditions, such as thyroid dysfunction, kidney or liver disease, or food allergies to black cumin seed. Patients who were unwilling to continue at any point, developed health conditions during the study, or showed evidence of an allergy to seed were excluded.

### 2.13 Interventions

The test group received an oral intervention of 5 g of black cumin seed powder daily for 8 weeks (5 g once every 24 h). The control group did not receive black cumin seed. Following the intervention, the serum lipid levels were analyzed to compare the changes in both groups before and after the intervention.

### 2.14 Ethical Considerations

The study design received approval from the Ethics Committee of Chattogram Veterinary and Animal Sciences University, Bangladesh, under the ethical code CVASU/Dir (R&E) EC/2022/435 (1)/4. Furthermore, written informed consent was obtained from all participants prior to the intervention.

### 2.15 Effect of Black Cumin Seed Intervention on CNAQ Scores

**FIGURE 2:**
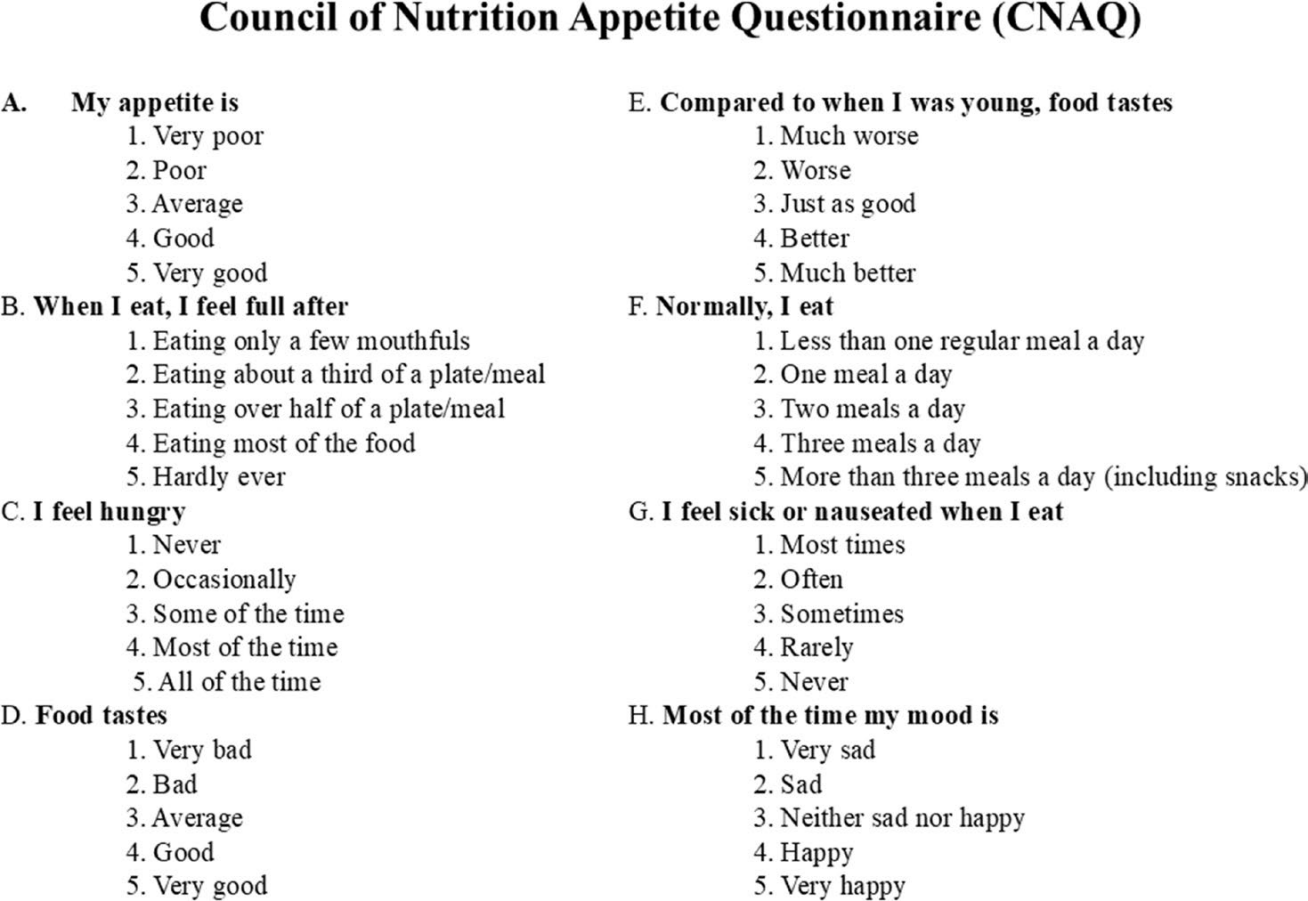
CNAQ scores among participants in a clinical trial evaluating the effects of black cumin seed powder intervention. The test group received black seed powder, while the control group did not.

Participants completed the validated English version of the CNAQ, an eight‐item instrument designed to assess appetite by evaluating factors such as feelings of fullness, hunger, food taste, changes in food taste, number of daily meals, nausea during eating, and usual mood (Wilson et al. 2005). Each item is rated on a five‐point Likert scale, with responses ranging from “A” to “E”. The total CNAQ score ranges from 8 (indicating the poorest appetite) to 40 (indicating the best appetite), calculated by summing the scores of all eight items (Figure 2). A lower total score signifies a diminished appetite. Completing the questionnaire typically takes about 5 min. All participants in both the test and control groups completed the questionnaire before and after the intervention. Those who consented signed a consent form and proceeded to complete the CNAQ.

### 2.16 Internal Consistency of CNAQ

The internal consistency assessment of the CNAQ involved calculating Cronbach's alpha coefficient to evaluate internal consistency. This coefficient was determined for both the test and control groups at pre‐intervention and post‐intervention stages. Individual item responses from all participants were collected at both time points, and Microsoft Excel 2010 was utilized to compute Cronbach's alpha using the following formula (Cronbach 1951):

$$\alpha =\frac{k}{k-1}\left(1-\frac{\sum_{i=1}^{k}\sigma^{2}y_{i}}{\sigma^{2}y}\right)$$

where, *k* is the number of items, $\sigma^{2}y_{i}$ is the variance of each item, and $\sigma^{2}y$ is the variance of the total score.

### 2.17 Blood Collection and Analysis of Serum Lipid

Fasting blood samples for serum lipid analysis were collected from the antecubital vein in the morning (8.00 AM–10.00 AM) after an overnight fast. A total of 10 mL of blood was collected into plain vacutainer tubes for subsequent analysis. The serum was separated by centrifuging the clotted blood at 3500 rpm for 10 min at 4°C, and then stored at −80°C for biochemical testing (Hadi et al. 2021). The lipid profile (total cholesterol (TC), LDL‐cholesterol (LDL‐C), HDL‐cholesterol (HLD‐C), and triglyceride (TG)) was measured using Rader's method (Rader 2022) on an automated analyzer at Karnafully Diabetic Center, Chattogram, Bangladesh.

### 2.18 Statistical Analysis

Data are expressed as mean ± SD (in vitro experiments) or mean ± SE (in vivo experiments). Statistical analyses were performed using GraphPad Prism version 9.5.1 (733). To assess statistical significance, analysis of variance (ANOVA) was conducted, followed by the Tukey–Kramer test for each experiment. Each experiment comprised three independent trials (*n* = 3). For the analysis of participant's CNAQ scores, the Mann–Whitney *U* test was employed, with a significance threshold set at *p* < 0.05. Paired sample *t*‐tests were conducted to determine the mean difference between before and after the intervention of serum sample measurements, with significance set at *p* < 0.01. An ANCOVA was performed to compare post‐intervention cholesterol levels between groups, using baseline cholesterol as a covariate and group assignment as a fixed factor. The Shapiro–Wilk test was performed to confirm the normality of residuals and outcome variables using GraphPad Prism version 9.5.1 (733).

## 3 Result

### 3.1 Phytochemicals in BSE Extract

**FIGURE 3:**
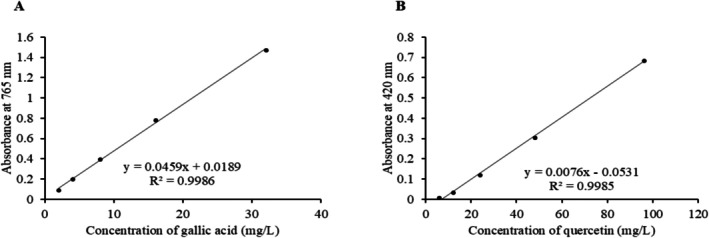
Calibration curves used for the quantification of phenolic and flavonoid compounds in black cumin seed extract. (A) Calibration curve of gallic acid standard for the determination of total phenolic content (TPC), expressed as mg gallic acid equivalents (GAE) per gram of dry weight. (B) Calibration curve of quercetin standard for the determination of total flavonoid content (TFC), expressed as mg quercetin equivalents (QE) per gram of dry weight.

The total phenolic content (TPC) of the black cumin seed extract, determined using the Folin–Ciocalteu method, was found to be 35.475 ± 0.065 mg GAE/g DW. The calibration curve for gallic acid showed good linearity (*R* ^2^ > 0.99) within the tested range (2–32 μg/mL) (Figure 3A). The total flavonoid content (TFC), measured by the aluminum chloride colorimetric method, was 39.51 ± 0.071 mg QE/g DW. The quercetin standard curve was also linear (*R* ^2^ > 0.99) across the concentration range of 6–96 μg/mL (Figure 3B). All values are presented as mean ± standard deviation (*n* = 3), indicating good reproducibility of the measurements.

### 3.2 Interpretation of FTIR Spectra

**FIGURE 4:**
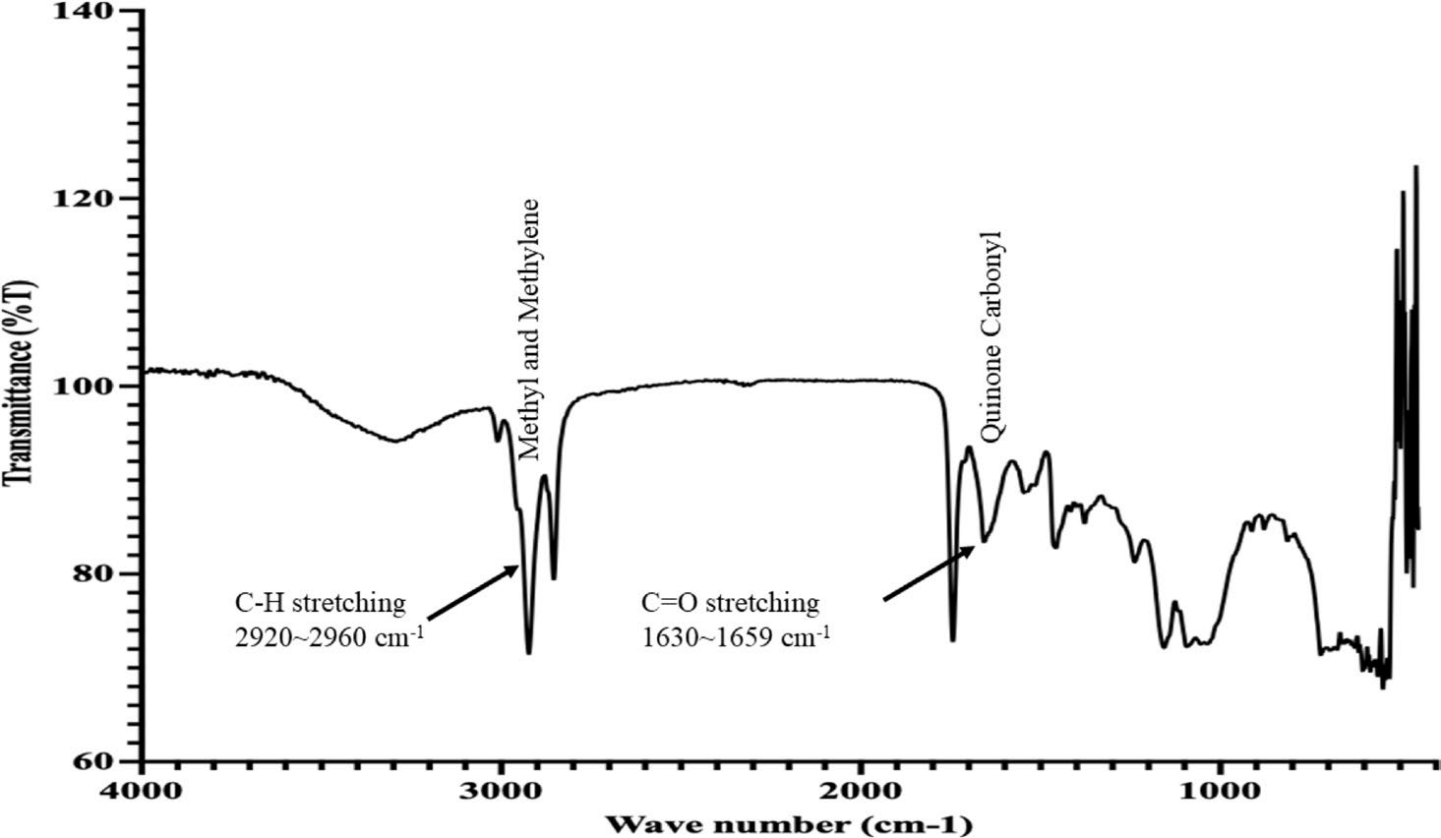
FTIR spectrum of black cumin seed extract showing characteristic peaks corresponding to various functional groups. Notably, an absorption band observed between 1630 and 1660 cm^−1^ indicates the presence of C=O stretching vibrations of the carbonyl group, confirming the presence of quinone structures such as thymoquinone. Other peaks represent functional groups typically found in phytochemical constituents of the extract.

The obtained FTIR spectra were analyzed to identify characteristic absorption bands corresponding to various functional groups (Figure 4). Particular attention was given to the identification of functional groups associated with thymoquinone, the key bioactive compound in black cumin seed. The presence of peaks near ~1660 cm^−1^ (C=O stretching of quinone), ~1590–1610 cm^−1^ (aromatic C=C stretching), and ~1260 cm^−1^ (C–O stretching or C–H bending of aromatic ring) was indicative of thymoquinone (Piras et al. 2013; Ahmad et al. 2013). Transmittance values were recorded for each relevant peak to support spectral interpretation.

### 3.3 Fatty Acid Profile by GC–MS

**FIGURE 5:**
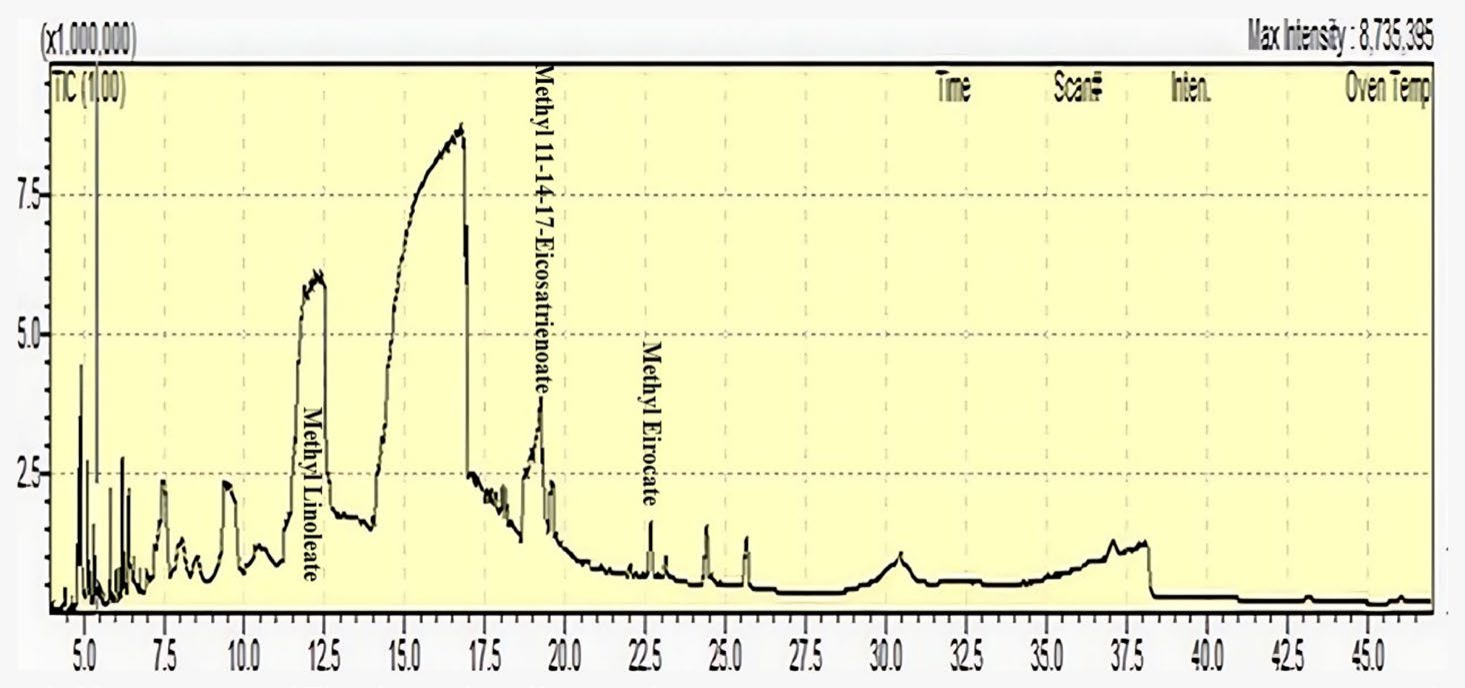
GC–MS chromatogram showing the fatty acid profile of black cumin seed. A total of 23 fatty acids were identified, each represented by distinct peaks in the spectrum. Among them, three fatty acids—methyl eicosatrienoate (2216.91 ± 103.57 ppm), methyl 11,14,17‐eicosatrienoate (822.91 ± 39.89 ppm), and methyl linoleate (129.47 ± 17.94 ppm)—were found in the highest concentrations, collectively accounting for the majority of total fatty acids. Peak intensities correspond to the relative concentrations of fatty acids in parts per million (ppm).

Gas Chromatography–Mass Spectrometry (GC–MS) analysis of black cumin seed oil from our seed sample identified approximately 23 different fatty acids. Among these, three fatty acids were present at significantly higher concentrations (Figure 5). Methyl eicosatrienoate was the most abundant, accounting for 69.29% of the total fatty acid content, with a concentration of 2216.91 ± 103.57 ppm. This was followed by methyl 11,14,17‐eicosatrienoate at 25.2%, corresponding to 822.91 ± 39.89 ppm. Methyl linoleate was detected at a lower concentration of 129.47 ± 17.94 ppm, representing 4.05% of the total fatty acids. These results indicate that methyl eicosatrienoate and methyl 11,14,17‐eicosatrienoate are the predominant fatty acids in black cumin seed oil.

### 3.4 Effects of BSE on 3T3‐L1 Preadipocyte Viability

**FIGURE 6:**
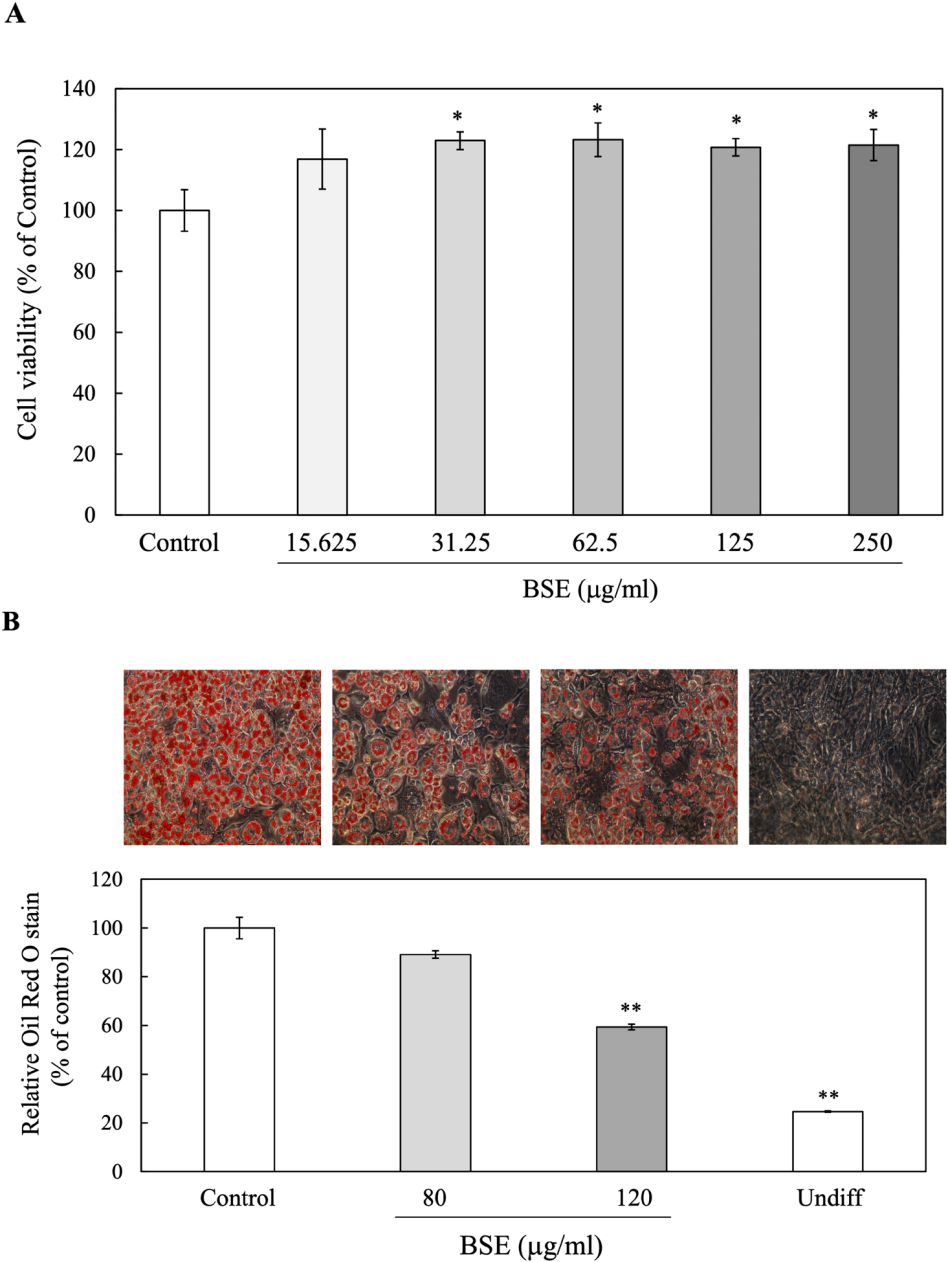
The effect of BSE on the viability and lipid accumulation of 3T3‐L1 preadipocytes was evaluated. Cells were treated with 80 or 120 μg/mL of BSE for 24 h. (A) Cell viability was determined using the neutral red assay. (B) Representative images of oil red O staining in 3 T3‐L1 cells. Intracellular lipid levels were measured through oil red O staining 8 days after initiating differentiation. Quantitative analysis was performed on 3T3‐L1 preadipocytes treated with 80 or 120 μg/mL of BSE, compared to untreated controls. Oil red O was extracted, and its absorbance was recorded at 520 nm using a spectrophotometer. Data are presented as means ± SD (*n* = 4). *p < 0.05, **p < 0.01 versus control.

The neutral red assay was employed to assess the viability of 3T3‐L1 preadipocytes when exposed to BSE. Notably, BSE concentrations up to 250 μg/mL did not exhibit any harmful cytotoxic effects on these cells (Figure 6A). Cell viability remained consistent with control groups across all tested concentrations, indicating that the extract is non‐cytotoxic to 3T3‐L1 cells within this range. Therefore, 80 μg/mL and 120 μg/mL were selected as the optimal concentrations for further experiments. These levels were chosen to maintain cell viability while ensuring adequate extract exposure to evaluate potential impacts on adipocyte differentiation.

### 3.5 Impact of BSE on Lipid Accumulation in 3 T3‐L1 Preadipocytes

In this study, confluent 3T3‐L1 preadipocytes were treated with various concentrations of BSE during MDI induction to evaluate its effect on lipid accumulation. Intracellular triglyceride accumulation was assessed using oil red O staining on the 8th day post‐differentiation initiation. The study observed that BSE effectively reduced lipid accumulation in cells with reductions of 10.9% (*p* = 0.1203) and 40.63% (*p* = 0.0082) at concentrations of 80 μg/mL and 120 μg/mL, respectively, compared to control cells (Figure 6B). These results suggest that BSE may impede adipocyte differentiation in 3T3‐L1 cells, indicating its potential efficacy in addressing obesity.

### 3.6 Impact of BSE on Glycerol‐3‐Phosphate Dehydrogenase Activity in 3T3‐L1 Preadipocytes

**FIGURE 7:**
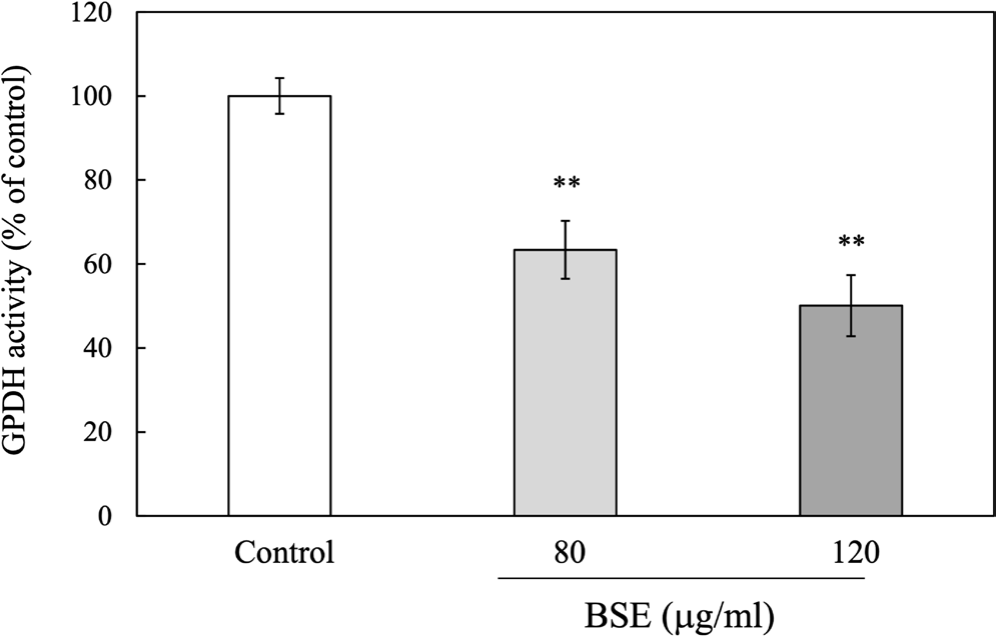
GPDH activity in 3T3‐L1 preadipocytes treated with BSE at 80 or 120 μg/mL. GPDH enzyme activity was measured based on the reduction in NADH levels every 3 min, using an extinction coefficient of 6.22 mM^−1^ cm^−1^. Enzyme activity is expressed as a percentage relative to the untreated control. Data are presented as means ± SD (*n* = 4). **p < 0.01 versus control.

GPDH is a cytosolic enzyme that catalyzes the reduction of dihydroxyacetone phosphate (DHAP) to glycerol 3‐phosphate, a key intermediate in triglyceride synthesis. This reaction is essential in lipid metabolism, as glycerol 3‐phosphate forms the backbone for triglyceride assembly. By facilitating this conversion, GPDH significantly influences the rate of triglyceride biosynthesis (Kuri‐Harcuch 1978). During the final stages of adipocyte differentiation and maturation, GPDH activity markedly increases (Wise and Green 1979). Our study aimed to assess GPDH activity to verify the inhibitory impact of BSE treatment on triglyceride synthesis. As shown in Figure 7, administering BSE at 80 μg/mL and 120 μg/mL reduced GPDH activity, which corresponded with a decline in triglyceride levels. These findings suggest that GPDH activity serves as a reliable marker for adipocyte differentiation and imply that BSE plays a significant role in adipogenesis by decreasing glycerol production in 3T3‐L1 cells.

### 3.7 Influence of BSE on the Expression of Adipogenic Key Regulators

**FIGURE 8:**
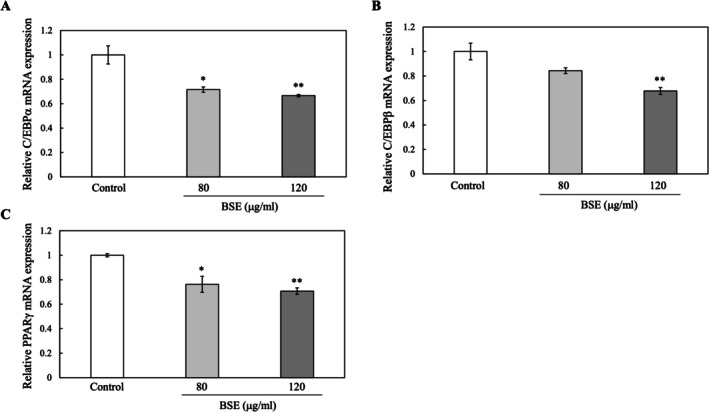
Effect of BSE on the mRNA expression levels of (A) C/EBPα, (B) C/EBPβ, and (C) PPARγ after 48 h of differentiation in 3T3‐L1 preadipocytes. Data are presented as mean ± SD (*n* = 3). *p < 0.05, **p < 0.01 versus control.

The process of adipogenesis is orchestrated by a network of transcription factors, with CCAAT/enhancer‐binding protein beta (C/EBPβ) playing a pivotal role. C/EBPβ is crucial in the early stages of adipocyte differentiation, initiating the expression of other key adipogenic regulators (Farmer 2006). Reducing the expression of CCAAT/enhancer‐binding protein beta (C/EBPβ) in 3T3‐L1 preadipocytes hampers the process of adipogenesis (Guo et al. 2015). C/EBPβ serves as a pivotal early‐stage regulator by binding to the promoters of key adipogenic transcription factors, specifically C/EBPα and peroxisome proliferator‐activated receptor gamma (PPARγ), thereby initiating their activation (Tang and Lane 1999). These transcription factors, PPARγ and C/EBPα, are indispensable in orchestrating the transcriptional network that facilitates the transformation from preadipocytes to mature adipocytes (Park et al. 2012). The activation of PPARγ during the differentiation of preadipocytes is crucial for adipogenesis; its absence impedes precursor cells from developing into adipocytes (Rosen et al. 2000). Notably, PPARγ can drive adipogenesis independently of C/EBPα, whereas C/EBPα alone is insufficient to trigger adipogenesis in the absence of PPARγ, highlighting PPARγ's role as the primary regulator of adipogenesis (Rosen et al. 2002). To further investigate the differentiation of 3T3‐L1 cells, quantitative real‐time PCR (qRT‐PCR) was utilized to assess mRNA expression levels. After 48 h of treatment with BSE, there was a significant reduction in the mRNA levels of C/EBPα, C/EBPβ, and PPARγ compared to the control group (Figure 8A–C). These findings indicate that BSE suppresses adipogenesis by downregulating the expression of these key transcription factors.

### 3.8 Demographic and Clinical Characteristics of Study Participants

Based on preliminary data, the effect size (Cohen's d) was estimated at 1.33, indicating a large treatment effect. Using G*Power with a significance level of 0.05 and power of 0.80, the required sample size was calculated as 36 (18 per group). To accommodate potential dropouts, 42 eligible participants were enrolled, with 22 in the treatment group and 20 in the control group. This sample size was considered adequate to assess the lipid‐lowering effects of black cumin seed powder. These participants were allocated into two groups: 22 in the experimental group and 20 in the control group. In the experimental group, 27.27% were women and 72.72% were men. Age distribution was 68.18% between 20 and 50 years old, and 31.82% over 50years old. All participants had a body mass index (BMI) exceeding 25.0 kg/m^2^. In the control group, 60% were women and 40% were men. Age distribution was 75% between 20 and 50 years old, and 25% over 50years old. Similarly, all participants had a BMI exceeding 25.0 kg/m^2^ (Table 2). These demographic and clinical characteristics are essential for understanding the study population and ensuring the applicability of the findings. Detailed reporting of such characteristics aligns with the CONSORT 2010 guidelines, which emphasize the importance of providing comprehensive baseline data to facilitate the interpretation and replication of clinical trials.

**TABLE 2:** Baseline characteristics of study participants.

Variables	Overall (*n* = 42)	Test group (*n* = 22)	Control group (*n* = 20)	Baseline *p* value
Age (year)	46.4	45.5	47.3	0.265
Weight (Kg)	74.8	75.6	73.9	0.208
Height (cm)	163.18	164.5	161.8	0.123
BMI (Kg/m^2^)	28.1	29.0	29.2	0.364

*Note:* Significance test (independent *t*‐test) was conducted to compare baseline characteristics between the groups. The baseline *p* value indicated no significant difference between the groups.

### 3.9 Impact of Black Seed Supplementation on Appetite as Measured by the Council on Nutrition Appetite Questionnaire

In this randomized controlled trial, we assessed the effect of an 8‐week black cumin seed supplementation on appetite among participants, using the CNAQ as the assessment tool. The intervention group (*n* = 22) experienced a significant increase in CNAQ scores over the 8‐week period (pre‐intervention: 27.64 ± 1.65; post‐intervention: 28.68 ± 1.29; *p* < 0.05), indicating no overall adverse effects of black cumin seed on appetite. Conversely, the control group (*n* = 20) showed no significant change in CNAQ scores (pre‐intervention: 29 ± 1.89; post‐intervention: 28.25 ± 1.71; *p* = 0.1966), suggesting stable appetite levels (Table 3).

**TABLE 3:** CNAQ scores and Cronbach's alpha coefficient for reliability assessment in a trial evaluating the effects of black seed powder intervention.

Period of intervention	CNAQ score		Cronbach's alpha	
	Control	Test	Control	Test
Before	29 ± 1.89	27.64 ± 1.65	0.64	0.86
After	28.25 ± 1.71	28.68 ± 1.29	0.70	0.94

*Note:* CNAQ score indicated mean ± SE (Control group, *n* = 20; Test group, *n* = 22).

### 3.10 Impact of Black Cumin Seed Supplementation on the Internal Consistency of CNAQ

The internal consistency of the CNAQ was assessed using Cronbach's alpha coefficient at two time points—before and after the 8‐week intervention—in both the test group (black cumin supplementation) and the control group (no supplementation). In the control group, Cronbach's alpha was 0.64 at baseline and increased to 0.70 post‐intervention, indicating a shift from questionable to acceptable internal consistency. In the test group, the coefficient was 0.86 before the intervention and increased to 0.94 after the intervention, suggesting a move from good to excellent internal consistency (Table 3). These results indicate that the CNAQ showed acceptable to excellent internal consistency across both groups at both time points. However, changes in Cronbach's alpha reflect variation in internal consistency and should not be interpreted as direct evidence of biological or behavioral effects of the intervention.

### 3.11 Impacts of Black Cumin Seed Supplementation on Serum Cholesterol

**FIGURE 9:**
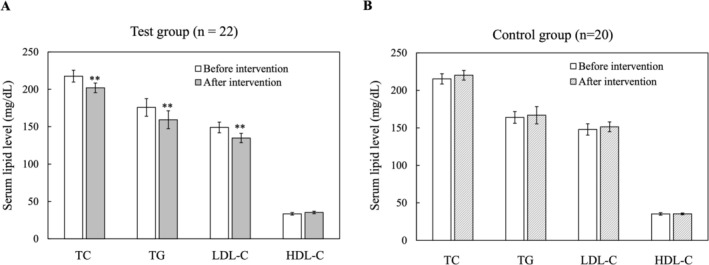
Effects of black cumin seed supplementation on serum lipid profiles over an 8‐week intervention period. Participants were randomized into a control group (*n* = 20) with no intervention and a test group (*n* = 22) receiving black cumin seed supplementation. Baseline lipid profiles were comparable between groups. Data are presented as mean ± SE. ** *p* < 0.01 relative to the control.

In this experimental trial involving black cumin seed supplementation, participants exhibited no signs of clinical toxicity and did not experience any significant adverse effects or side effects. In our randomized controlled trial, we evaluated the impact of black cumin seed supplementation on serum lipid profiles over an 8‐week period. Participants were randomly assigned to either a control group (*n* = 20) receiving no intervention or a test group (*n* = 22) receiving black cumin seed supplementation. Baseline measurements indicated comparable lipid profiles between the two groups. Post‐intervention analysis revealed that the test group experienced significant reductions in total cholesterol (from 217.61 ± 7.93 mg/dL to 201.95 ± 6.46 mg/dL), LDL‐C (from 149 ± 7.14 mg/dL to 134.82 ± 6.2 mg/dL), and TG (from 175.77 ± 11.77 mg/dL to 159.27 ± 11.92 mg/dL), along with an increase in HDL‐C (from 33.45 ± 1.72 mg/dL to 35.27 ± 1.65 mg/dL) (Figure 9A). In contrast, the control group showed no significant changes in lipid parameters (Figure 9B). Paired *t*‐tests confirmed that black cumin seed supplementation led to significant improvements in serum lipid profiles, suggesting its potential as an effective adjunct therapy for dyslipidemia management.

## 4 Discussion

The phytochemical richness of black cumin seed underlies its diverse pharmacological properties, including antioxidant, anti‐inflammatory, and anti‐adipogenic effects (Ahmad et al. 2013). In this study, high levels of total phenolic content (35.475 ± 0.065 mg GAE/g DW) and total flavonoid content (39.51 ± 0.071 mg QE/g DW) highlight its strong antioxidant potential. These metabolites are known for their free radical scavenging capacity and regulatory effects on lipid metabolism (Dudonné et al. 2009). FTIR analysis confirmed the presence of functional groups linked to thymoquinone, with characteristic peaks at ~1660 cm^−1^ (C=O), ~1590–1610 cm^−1^ (C=C), and ~1260 cm^−1^ (C–O/C–H), consistent with previous studies (Piras et al. 2013; Ahmad et al. 2013). These findings indicate a rich presence of aromatic and phenolic compounds that may contribute to the extract's bioactivity. GC–MS profiling revealed 23 fatty acids, predominantly methyl eicosatrienoate (69.29%), methyl 11,14,17‐eicosatrienoate (25.2%), and methyl linoleate (4.05%). The dominance of unsaturated fatty acids reflects the nutritional and therapeutic value of black cumin seed, particularly due to PUFAs known for lipid‐lowering and anti‐inflammatory actions (Cheikh‐Rouhou et al. 2007). Together, the phytochemical, FTIR, and fatty acid data support the potential of black cumin seed as a functional food with therapeutic relevance, especially in metabolic health contexts.

The assessment of 3T3‐L1 preadipocyte viability following treatment with BSE showed no significant cytotoxic effects at concentrations up to 250 μg/mL, as cell viability remained similar to the control group. This is consistent with previous studies where black cumin seed extracts showed low cytotoxicity in various cell lines, including fibroblasts and adipocytes, at similar concentrations (Bashir et al. 2023; Bordoni et al. 2019; Mashayekhi‐Sardoo et al. 2020). The absence of cytotoxicity at these concentrations underscores the potential of BSE as a safe natural agent for further exploration in adipogenesis research. Based on these observations, concentrations of 80 μg/mL and 120 μg/mL were selected for subsequent experiments. These concentrations strike a balance between ensuring sufficient exposure for evaluating biological activity and preserving cellular viability, a critical consideration in studies targeting adipocyte differentiation.

The findings of this study demonstrate that BSE effectively reduces lipid accumulation in 3T3‐L1 preadipocytes during adipocyte differentiation. Specifically, BSE treatment at concentrations of 80 μg/mL and 120 μg/mL led to reductions in intracellular triglyceride accumulation by 10.9% (*p* = 0.1203) and 40.63% (*p* = 0.0082), respectively, compared to the control. The significant reduction in lipid accumulation observed at 120 μg/mL aligns with a previous study reporting the anti‐adipogenic properties of bioactive compounds in black cumin seed, particularly thymoquinone, which has been shown to regulate lipid metabolism through the modulation of transcription factors involved in adipogenesis, such as PPARγ and C/EBPβ (Ahmed et al. 2025). The modest reduction at 80 μg/mL, though not statistically significant, suggests that lower concentrations may require prolonged exposure or co‐treatment with other bioactive compounds to enhance efficacy. Additionally, these findings support the potential application of BSE as a natural therapeutic agent in obesity management. Unlike synthetic anti‐obesity drugs, which often come with adverse effects (Kang and Park 2012), plant‐based extracts like BSE offer a safer alternative with minimal side effects (Saad 2023). However, in vivo studies and clinical trials are warranted to establish the extract's safety, bioavailability, and efficacy in human subjects.

This study examines the impact of BSE on GPDH activity in 3T3‐L1 preadipocytes, focusing on its potential role in inhibiting adipogenesis. GPDH, an enzyme crucial in lipid metabolism, facilitates the conversion of DHAP to glycerol‐3‐phosphate, a precursor for triglyceride synthesis (Kuri‐Harcuch 1978). The increase in GPDH activity during the terminal stages of adipocyte differentiation reflects its pivotal role in triglyceride accumulation (Wise and Green 1979). Our results show that BSE treatment significantly reduces GPDH activity at 80 μg/mL and 120 μg/mL, leading to lower triglyceride accumulation. This suggests that BSE inhibits adipogenesis by targeting GPDH, thus impeding fat storage. These findings support previous research that downregulating GPDH disrupts adipocyte differentiation and lipid accumulation (Green and Kehinde 1975; Zebisch et al. 2012). This aligns with reports of other natural compounds that inhibit adipocyte differentiation by targeting enzymes involved in triglyceride synthesis (Rayalam et al. 2008). In conclusion, BSE appears to be a potent natural inhibitor of adipogenesis, warranting further investigation for potential anti‐obesity applications.

This study shows that BSE effectively inhibits adipogenesis in 3T3‐L1 preadipocytes, especially during early differentiation stages. A 46.22% reduction in lipid accumulation with 120 μg/mL BSE aligns with previous research on plant‐derived compound's ability to hinder adipocyte differentiation (Haselgrübler et al. 2019; Wong et al. 2014). BSE may interfere with key signaling pathways involved in adipocyte commitment and maturation, likely by modulating transcription factors like C/EBPβ and PPARγ, crucial for adipogenic gene expression (Lee and Ge 2014). This inhibition prevents the activation of downstream targets necessary for lipid droplet formation. These results suggest BSE's potential in managing obesity‐related metabolic disorders. Further research is needed to identify active compounds and investigate molecular mechanisms in vivo. In conclusion, BSE demonstrates strong anti‐adipogenic effects, laying the foundation for exploring its therapeutic applications in metabolic diseases.

The present study examines how BSE affects key adipogenic regulators, specifically C/EBPβ, C/EBPα, and PPARγ, which are crucial for adipogenesis. Previous research has shown that C/EBPβ initiates adipocyte differentiation by activating downstream factors like C/EBPα and PPARγ (Farmer 2006; Tang and Lane 1999). Our results indicate significant downregulation of these transcription factors after BSE treatment, suggesting that BSE disrupts adipogenesis by impairing this regulatory network. Consistent with Guo et al. (2015), who reported that reduced C/EBPβ expression halted adipocyte differentiation in 3T3‐L1 preadipocytes, our study shows a similar reduction in C/EBPβ expression following BSE treatment. Furthermore, PPARγ and C/EBPα are essential for complete adipocyte differentiation (Park et al. 2012). Notably, our findings demonstrate a significant decrease in PPARγ expression after BSE treatment, aligning with Rosen et al. (2000) identification of PPARγ as a key regulator in adipocyte maturation. Interestingly, our study shows that BSE treatment significantly decreases PPARγ expression, aligning with the findings of Rosen et al. (2002) which emphasizes PPARγ's crucial role in adipogenesis. While C/EBPα can drive differentiation with PPARγ, it cannot induce adipogenesis alone. Therefore, PPARγ downregulation is key to BSE's suppression of adipocyte formation. Overall, our results suggest that BSE inhibits adipogenesis by downregulating essential transcription factors, including C/EBPβ, C/EBPα, and PPARγ, disrupting the pathways required for adipocyte differentiation.

In this study, the demographic and clinical characteristics of participants provide insights into the generalizability of the findings. A total of 42 individuals participated, with 22 in the experimental group and 20 in the control group. The experimental group had a higher proportion of males (72.72%) compared to the control group, which had 60% females, consistent with recruitment strategies observed in other studies (Merone et al. 2022). Most participants were aged 20–50 years, with 68.18% in the experimental group and 75% in the control group. This aligns with other research targeting adults in this age range (Wang et al. 2023; Zhang et al. 2023). The inclusion of participants over 50 years (31.82% in the experimental group and 25% in the control group) ensures the study accounts for age‐related differences in metabolic response. All participants had a BMI above 25.0 kg/m^2^, indicating they were overweight or obese, a group at higher risk for metabolic disorders (Van Cauwenberge et al. 2024). The study's reporting of demographic and clinical characteristics follows CONSORT 2010 guidelines, ensuring transparency and reproducibility in clinical trials (Moher et al. 2009; Schulz et al. 2010).

In this study, we assessed the effect of black cumin seed supplementation on appetite using the CNAQ. Results showed a significant improvement in appetite in the intervention group, with CNAQ scores increasing from 27.64 ± 1.65 to 28.68 ± 1.29 (*p* < 0.05). This aligns with prior studies suggesting that black cumin seeds, rich in bioactive compounds, can enhance appetite regulation, particularly through thymoquinone, which has anti‐inflammatory and appetite‐modulating effects (Khader and Eckl 2014). The control group, which did not receive supplementation, showed no significant change (*p* = 0.1966), confirming the effects were due to the supplementation. These findings suggest black cumin seed supplementation may benefit individuals with appetite‐related issues, such as those with reduced appetite due to illness or weight loss (Al Asoom 2022). Moreover, our results align with previous findings where herbal supplements, particularly those with anti‐inflammatory and antioxidant properties (Mashmoul et al. 2013), have been shown to influence appetite regulation in clinical trials. Black cumin seed's effect on appetite may be a result of its ability to influence the central nervous system, as seen in animal studies with other similar botanicals (Beheshti et al. 2016).

The primary aim of this study was to evaluate the impact of black cumin seed supplementation on the internal consistency of the CNAQ. Results showed a significant improvement in internal consistency, measured by Cronbach's alpha, in the group receiving black cumin. Before the intervention, the test group had a Cronbach's alpha of 0.86, indicating good internal consistency, consistent with previous studies on standardized questionnaires (Jones et al. 2015). After supplementation, the alpha coefficient increased to 0.94, suggesting excellent internal consistency, likely due to black cumin seed's anti‐inflammatory and antioxidant properties (Alberts et al. 2024). In contrast, the control group showed a smaller increase in Cronbach's alpha, from 0.64 to 0.70, indicating only moderate improvement. This suggests that black cumin supplementation likely contributed more to the observed effects in the test group. Overall, the study highlights the potential of black seed to improve the internal consistency of the CNAQ and supports its cognitive and health benefits in nutritional assessments.

This randomized controlled trial assessed the effect of black cumin seed supplementation on serum lipid profiles in healthy adults over 8 weeks. The results showed significant improvements in lipid parameters, including reductions in TC, LDL‐C, and TG, as well as an increase in HDL‐C. These findings suggest that black cumin seeds may help manage dyslipidemia and reduce cardiovascular risk. Al‐Naqeep et al. (2009) reported that oral administration of a thymoquinone‐rich methanolic extract or volatile oil from *Nigella sativa* to rats with hyperlipidemia significantly reduced hepatic HMGCoA reductase activity, alongside reductions in serum cholesterol and LDLC. For instance, a study by Hallajzadeh et al. (2020) demonstrated a significant reduction in serum TC and LDL‐C levels following supplementation with black cumin oil in hyperlipidemic individuals. Similar results have been reported by Heshmati et al. (2015) and Kaatabi et al. (2012), who found that black cumin seed supplementation helped reduce TG and TC levels in diabetic patients. The increase in HDL‐C following black cumin seed supplementation may further enhance its cardiovascular benefits, aligning with the findings of earlier studies by Rounagh et al. (2024), who reported similar HDL‐cholesterol elevating effects in individuals taking black seed. No significant changes were found in the control group, confirming the effectiveness of the intervention. Additionally, no adverse effects were observed, supporting the safety of black cumin seed supplementation, as noted by Hosseinzadeh et al. (2017) and Thomas et al. (2022). A key limitation of our study is the absence of a placebo in the control group, which may introduce bias. This decision was based on logistical and ethical constraints. To mitigate potential effects, both groups received the same dietary and lifestyle guidance, participants were single‐blinded, and outcome assessments were conducted by blinded investigators. Nonetheless, the lack of a placebo may still influence subjective outcomes and should be considered when interpreting the results.

In summary, the results of this study indicate that supplementation with lack cumin seed may substantially enhance serum lipid profiles, offering potential benefits for managing dyslipidemia and reducing cardiovascular risk. Due to these promising outcomes, additional long‐term research with larger sample sizes employing a double‐blind, placebo‐controlled design is necessary to validate these effects and investigate the underlying mechanisms.

## 5 Conclusion

The study highlights the potential of BSE in improving lipid metabolism. Both in vitro and human trials demonstrated its anti‐adipogenic and lipid‐lowering effects. In vitro, BSE reduced lipid accumulation and downregulated adipogenic transcription factors, suggesting interference with adipogenesis. The human trial showed improved lipid profiles, with lower serum TG, LDL‐C, and TC, and higher HDL‐C. These results suggest that black cumin seed ( *Nigella sativa* ) may serve as a promising natural agent in obesity‐related conditions, although further investigation involving comprehensive metabolic parameters is warranted.

## Author Contributions

**Shamima Ahmed:** conceptualization (lead), data curation (equal), investigation (lead), methodology (equal), writing – original draft (lead), writing – review and editing (lead). **Mohammad Shaokat Ali:** data curation (equal), formal analysis (lead), writing – original draft (equal), writing – review and editing (equal). **Yuki Nishigaki:** conceptualization (equal), investigation (equal). **Ranita Das:** investigation (equal), methodology (equal). **Sumsuddin Ahmed Shiblu:** methodology (equal), resources (equal). **Sharmin Akter:** formal analysis (equal), resources (equal). **Isao Matsui‐Yuasa:** visualization (supporting), writing – original draft (equal), writing – review and editing (equal). **Akiko Kojima‐Yuasa:** conceptualization (equal), investigation (equal), methodology (equal), project administration (lead), visualization (lead), writing – original draft (equal), writing – review and editing (lead).

## Conflicts of Interest

The authors declare no conflicts of interest.

## Supporting information


**Table S1:** fsn370888‐sup‐0001‐TableS1.pdf.

**Table S2:** fsn370888‐sup‐0002‐TableS2.pdf.

## Data Availability

The data that support the findings of this study are available from the corresponding author upon reasonable request.
