# Supplementary material for: Black Cumin Seed (Nigella sativa) Confers Anti‐Adipogenic Effects in 3T3‐L1 Cellular Model and Lipid‐Lowering Properties in Human Subjects
Source: Food Sci Nutr. 2025 Sep 1;13(9):e70888. doi: 10.1002/fsn3.70888 (PMC12401715; doi:10.1002/fsn3.70888)
Supplement: Supplementary file 1 — Table S1: fsn370888‐sup‐0001‐TableS1.pdf. [file FSN3-13-e70888-s002.pdf]

Total Phenolic Content (TPC)

Concentration of gallic acid (mg/L)	Absorbance
2	0.089
4	0.199
8	0.397
16	0.783
32	1.471

Rep1	7.108	0.329
Rep2	7.095	0.329
Rep3	7.082	0.328
Mean	7.095	
SD	0.013	

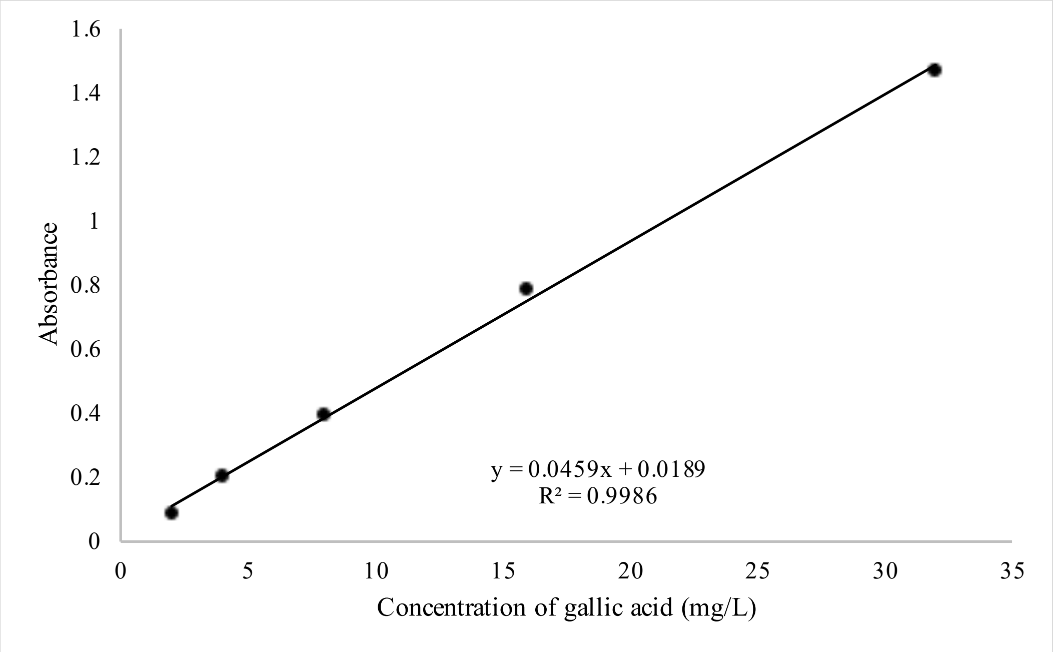

Rep1	35.54
Rep2	35.475
Rep3	35.41
Mean	35.475
SD	0.065

Using Dilution Factor = Final volume/Aliquot volume

5

Total TPC Concentration = Mean TPC *DF(5) = 35.475 mg/L

TPC (mg GAE/g DW) = TPC (mg GAE/L)*Vol of extract/Weight of sample

35.475 0.01 0.01

Total Flavonoid Content (TFC)

Concentration of quercetin (mg/L)	Absorbance
6	0.009
12	0.033
24	0.122
48	0.305
96	0.686

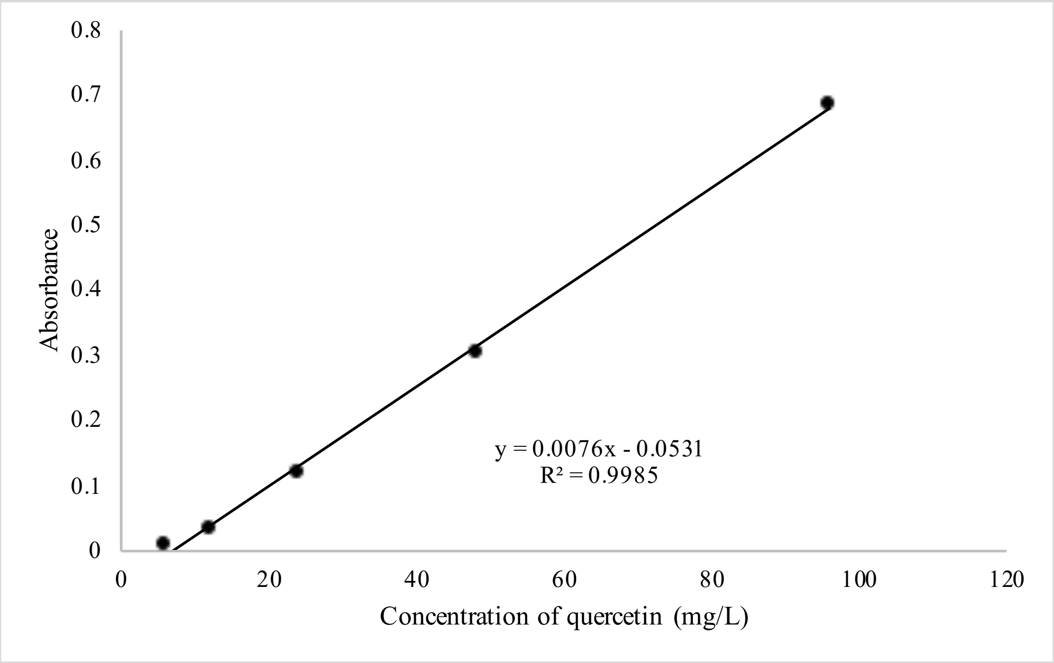

Rep1	39.5738	0.2967
Rep2	39.4296	0.2956
Rep3	39.5213	0.2963
Mean	39.50823333	
SD	0.072982624	

Rep1	395.738
Rep2	394.296
Rep3	395.213
Mean	395.0823333
SD	0.729826235

Using Dilution Factor = Final volume/Aliquot volume

10

Total TPC Concentration = Mean TPC *DF(5) = 395.0823 mg/L

TPC (mg GAE/g DW) = TPC (mg GAE/L)*Vol of extract/Weight of sample

395.0823 0.01 0.1

39.51
