# Supplementary material for: Black Cumin Seed (Nigella sativa) Confers Anti‐Adipogenic Effects in 3T3‐L1 Cellular Model and Lipid‐Lowering Properties in Human Subjects
Source: Food Sci Nutr. 2025 Sep 1;13(9):e70888. doi: 10.1002/fsn3.70888 (PMC12401715; doi:10.1002/fsn3.70888)
Supplement: Supplementary file 2 — Table S2: fsn370888‐sup‐0002‐TableS2.pdf. [file FSN3-13-e70888-s001.pdf]

	Name of fatty acid	Concentration of fatty acids (ppm)							
		Rep 1	Rep 2	Rep 3	Mean	SD	SEM	%	RT
1	Methyl Octanoate (111-11-5)	0.478	0.497	0.462	0.479	0.017521	0.010116	0.014972	5.414
2	Methyl Decanoate (110-42-9)	0.028	0.021	0.004	0.017667	0.012342	0.007126	0.000552	6.644
3	Methyl Laurate (111-82-0)	0.002	0.003	0.005	0.003333	0.001528	0.000882	0.000104	7.82
4	Methyl Tridecanoate (1731-88-0)	0.01	0.021	0.033	0.021333	0.011504	0.006642	0.000667	8.49
5	Methyl Myristate (124-10-7)	0.073	0.266	0.045	0.128	0.120329	0.069472	0.004001	9.28
6	Methyl Palmitpleate (1120-25-8)	0.003	0.171	0.035	0.069667	0.089204	0.051502	0.002177	11.165
7	Methyl Palmitate (112-39-0)	4.728	6.683	5.113	5.508	1.035628	0.59792	0.172157	11.417
8	Methyl Linoleate (112-63-0)	165.321	110.27	112.827	129.4727	31.07188	17.93936	4.046771	11.855
9	Methyl Oleate (112-62-9)	0.299	0.119	0.049	0.155667	0.12897	0.074461	0.004865	12.635
10	Methyl Linolenate (301-00-8)	0.023	0.031	0.006	0.02	0.012767	0.007371	0.000625	13.957
11	Methyl Stearate (112-61-8)	1.698	0.21	0.267	0.725	0.843125	0.486778	0.02266	14.137
12	Methyl Arachidate (1120-28-1)	0.576	0.113	0.06	0.249667	0.283853	0.163882	0.007804	14.505
13	Methyl Arachidonate (2566-89-4)	2.183	3.532	3.643	3.119333	0.812786	0.469262	0.097497	17.005
14	Methyl Eicosapaennoate (2734-47-6)	0.88	0.692	0.273	0.615	0.310739	0.179405	0.019222	17.089
15	Methyl 11-14-17-Eicosatrienoate (55682-88-7)	745.14	846.398	877.2	822.9127	69.09147	39.88998	25.72079	17.97
16	Methyl Heptadecanoate (1731-92-6)	9.208	12.335	11.561	11.03467	1.628589	0.940266	0.344897	18.77
17	Methyl Heneicosanoate (6064-90-0)	0.854	0.541	0.536	0.643667	0.182171	0.105177	0.020118	18.724
18	Methyl Docosapentaenoate (108698-02-8)	0.011	0.047	0.005	0.021	0.022716	0.013115	0.000656	21.745
19	Methyl Eicosatrienoate (1120-34-9)	2265.012	2367.347	2018.367	2216.909	179.394	103.5732	69.29124	22.274
20	Methyl 11-Eicosenponoate (2390-09-2)	0.182	0	0	0.060667	0.105078	0.060667	0.001896	23.722
21	Methyl Hehenate (929-77-1)	6.326	8.302	6.751	7.126333	1.040096	0.6005	0.222739	24.726
22	Methyl Tricosanoate (2433-97-8)	0.168	0	0	0.056	0.096995	0.056	0.00175	29.815
23	Methyl Nervonate (2733-88-2)	0.177	0	0	0.059	0.102191	0.059	0.001844	33.958
	Total concentration				3199.407				
